# Assessing the Agreement Between Diffusion Tension Imaging (DTI) and T2-Weighted MRI Sequence for Biometry of the Fetal Corpus Callosum

**DOI:** 10.3390/diagnostics14232700

**Published:** 2024-11-29

**Authors:** Liel N. Cohn, Shai Bookstein, Tamar Laytman Klein, Nadia Mordenfeld Kozlovsky, Tomer Ziv-Baran, Arnaldo Mayer, Eldad Katorza

**Affiliations:** 1 Arrow Program for Medical Research Education, Chaim Sheba Medical Center, Tel-Hashomer 5262000, Israel; lielcohn@mail.tau.ac.il (L.N.C.); bookstein1@mail.tau.ac.il (S.B.); nadia.mordenfeld@sheba.health.gov.il (N.M.K.); zivtome@tauex.tau.ac.il (T.Z.-B.); 2 School of Medicine, Faculty of Medical and Health Sciences, Tel Aviv University, Tel Aviv 6997801, Israel; arnaldo.mayer@sheba.health.gov.il; 3 Department of Oncology, Chaim Sheba Medical Center, Tel-Hashomer 5262000, Israel; 4 Department of Epidemiology and Preventive Medicine, School of Public Health, Faculty of Medical and Health Sciences, Tel Aviv University, Tel Aviv 6997801, Israel; 5 Department of Diagnostic Radiology, Chaim Sheba Medical Center, Tel-Hashomer 5262000, Israel; 6 Department of Obstetrics and Gynecology, Chaim Sheba Medical Center, Tel-Hashomer 5262000, Israel; 7 Gertner Institute for Epidemiology & Health Policy Research, Chaim Sheba Medical Center, Tel-Hashomer 5262000, Israel

**Keywords:** diffusion tension imaging (DTI), magnetic resonance imaging (MRI), T2 weighted sequence, fetal corpus callosum, fetal imaging

## Abstract

Background/Objectives: Little is known about the advantages of Diffusion Tensor Imaging (DTI) when evaluating the fetal corpus callosum (CC), a sensitive indicator for normal brain development. This study evaluates the contribution of DTI compared to T2-weighted imaging to assess fetal CC biometry. Methods: Data from the fetal MRI exams of singleton pregnancies between July 2017 and 2019 were retrospectively analyzed. Mid-sagittal sections were used to measure the CC biometry, and inter- and intra-observer agreements were assessed using the interclass correlation coefficient (ICC), targeting an ICC above 0.85. Results: The results from 100 patients (mean gestational age, 32.24 weeks) indicated excellent inter-observer reliability for DTI (ICC = 0.904, 95% CI = 0.815–0.952) and moderate agreement for T2-weighted imaging (ICC = 0.719, 95% CI = 0.556–0.842). Intra-observer assessments showed excellent reliability for both DTI and T2-weighted imaging (ICC = 0.967, 95% CI = 0.933–0.984 and ICC = 0.942, 95% CI = 0.884–0.971, respectively). However, a comparison between DTI and T2-weighted images for CC biometry showed poor agreement (ICC = 0.290, 95% CI = 0.071–0.476). Conclusions: In conclusion, the study highlights a lack of agreement between DTI and T2-weighted imaging in fetal CC biometry, suggesting the need for further research to understand this discrepancy and the role of DTI in fetal health.

## 1. Introduction

The corpus callosum (CC) is the largest interhemispheric commissure and plays a vital role in cognitive function [1]. The CC begins developing as early as 10 weeks’ gestation and becomes fully visible on transvaginal sonograms by 18 weeks’ gestation [2,3]. Disruptions in CC development can result in anomalies, such as partial or complete agenesis, hypoplasia, and hyperplasia, each with varying clinical implications [2,4]. However, diagnosing dysgenesis of the CC is further complicated by the dependence on gestational age-based reference charts for CC length, which are influenced by variations in population, sex, and personal growth variations, ultimately limiting the accuracy of these general CC length charts [5]. As a sensitive marker of brain development, the integrity of the CC during gestation can indicate the presence of additional abnormalities, particularly within the central nervous system (CNS) [6,7,8,9]. For instance, Greenbaum et al. found that fetal CC anomalies correlate with clinically significant chromosomal microarray analysis (CMA) findings [10]. Another study on children with partial agenesis of the CC found that while a majority of patients experience a favorable long-term prognosis, many still suffer from mild to severe disabilities, including speech disorders at school age and behavioral or motor deficits later in life [8]. Therefore, accurate visualization and diagnosis of the fetal CC are crucial, impacting prognosis, guiding clinical decision-making, and supporting informed parental counseling.

While ultrasonography (US) remains the primary tool for the initial assessment of the fetal CNS anatomy due to its accessibility and real-time imaging [11,12,13], its effectiveness is limited for midline structures, such as the CC [14]. Magnetic resonance imaging (MRI), particularly T2-weighted imaging, serves as an instrumental complementary tool following the detection of abnormalities in US [15,16] due to its high precision and improved diagnostic accuracy in the fetal brain, regardless of fetal presentation [17,18,19]. Comparative studies have exhibited varied results regarding the efficacy of MRI versus US for measuring the fetal CC. A recent study demonstrated that 3D super-resolution reconstructed (SR) T2-weighted MRI measurements of central sections of the CC closely match those obtained with US, noting some discrepancies in measurements of the anterior CC [20], while another study reported moderate agreement between US and conventional MRI [21]. A separate study demonstrated that MRI improves the detection and classification of CC anomalies compared to US, significantly influencing the clinical management of 35.9% of cases [22]. Lastly, Tilea et al. compared MRI with US biometry of the CC, demonstrating excellent agreement and providing useful reference charts for cerebral MRI biometry between 26 and 40 weeks’ gestation [15]. Despite the advantages of both US and conventional MRI in fetal CNS imaging, certain limitations still exist impacting the accuracy of the results including an incomplete understanding of prenatal brain maturation, incorrect MRI sequence selection, and artifacts [23].

To address these gaps, a relatively new MRI technology, diffusion tensor imaging (DTI), has emerged as a powerful tool by enhancing the visualization and characterization of white matter pathways with high specificity [24,25,26]. Previous research has demonstrated that DTI can effectively identify various types of CC abnormalities, such as CC agenesis, in both post-natal and prenatal imaging by accurately remodeling fiber-bundles and displaying the presence of Probst bundles (PB) otherwise not seen [27,28,29,30]. Therefore, integrating DTI into prenatal CC assessments could assist in overcoming the limitations in diagnosing fetal CC abnormalities and thereby providing parents with the necessary information regarding their future child’s prognosis [24,28,31]. The aim of our study is to evaluate the potential contribution of DTI in assessing the biometry of the fetal CC by evaluating the degree of agreement between DTI and MRI in the T2-weighted sequence.

## 2. Materials and Methods

### 2.1. Subjects

This retrospective observational study was approved by the institutional review board (IRB) of our medical center. The patient data were collected from fetal MRI examinations performed at the Sheba Medical Center, between July 2017 to July 2019. The inclusion criteria were as follows: (a) singleton pregnancy; (b) CC biometry and morphology reported as normal/absent of pathology; (c) imaging using a Phillips 3T MR machine (Philips Medical Systems, Best, The Netherlands) to reduce the potential sources of variability; (d) images exhibiting the entire biometry of the CC.

Patients were excluded if they had: (a) multiple pregnancy; (b) evidence of pathology in the fetus (or pathological MRI result); (c) imaging conducted using a non-Phillips MRI machine; (d) incomplete depiction of the biometry of the CC. Only satisfactory images with respect to quality and alignment were selected to be measured.

### 2.2. MR Imaging Technique

Based on our hospital protocol, mothers refrained from consuming sugary foods or drinks for a period of 4 h prior to the MRI examination. DTI and T2-weighted sequence were conducted consecutively during the same imaging session to reduce the risk of changes in fetal position and characteristics [32].

The MRI scans were acquired with a 3T magnet (Ingenia 3T, Philips Medical Systems, Best, The Netherlands)) under the following imaging parameters: single-shot fast spin-echo T2-weighted acquisition in sagittal plane, with a voxel size of 0.85 mm × 0.85 mm × 3 mm (no gap). TE and TR were set to 70 ms and 2545 ms, respectively. Following the T2-weighted sequence, a 12 directions Diffusion-Weighted Imaging (DWI) sequence was acquired in the sagittal plane (voxel size = 1.56 mm × 1.56 mm × 3 mm, no gap, b value = 800, and one reference scan at b-value = 0).

### 2.3. Measurements

**Figure 1:**
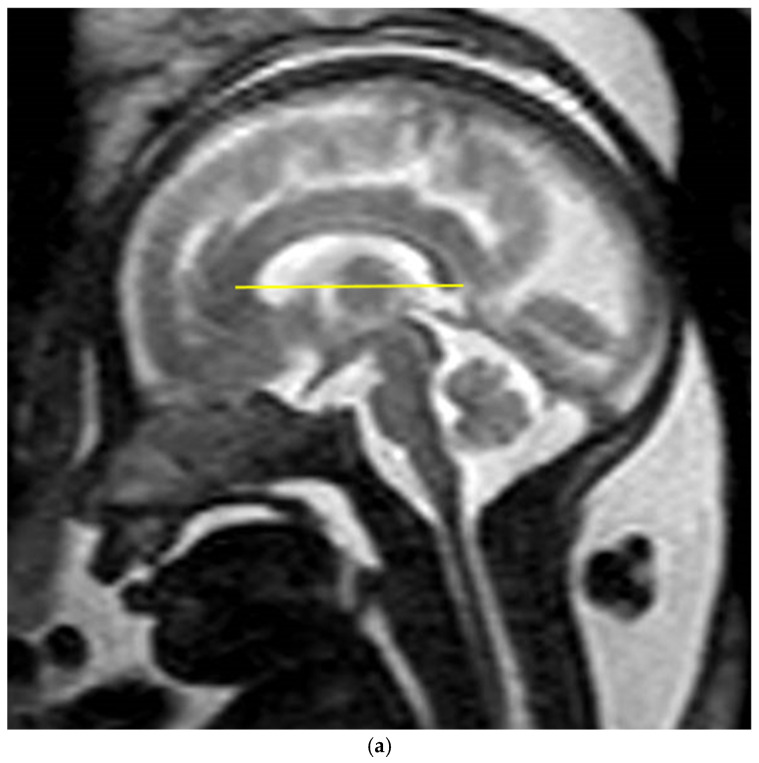

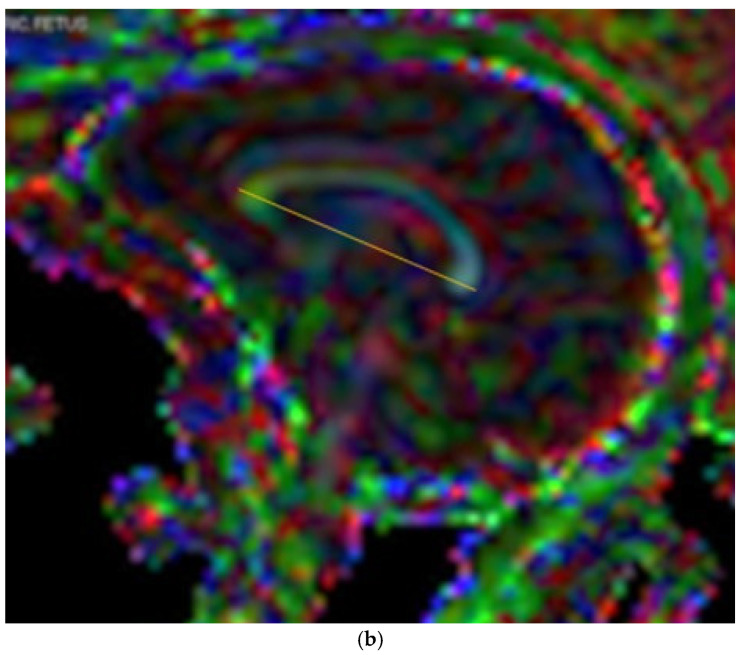
Mid-sagittal slice of the fetal brain in (**a**) T2-weighted image and (**b**) DTI with an example of fetal CC biometry measurement demonstrated by the yellow line. The images were taken from two different fetuses.

Mid-sagittal sections were used to assess and measure the biometry of the CC. Measurements were conducted in accordance with reference charts for fetal cerebral MRI biometry [15], using a straight rostrocaudal line from the most anterior aspect of the genu to the most posterior end of the splenium, parallel to the axial axis of the head (Figure 1a,b) [33,34]. Among the multiple MRI images available for each fetus, the image chosen for the measurement was based on the best mid-sagittal section, in which the brainstem and cerebellum were visible [33].

Three observers independently measured a cohort of 30 patients with images in both the T2-weighted sequence and DTI in order to establish inter-observer reliability of the measurement technique [35]. An additional reliability assessment was conducted with a single observer who measured the same cohort on two separate occasions to establish intra-observer reliability. Next, the same single observer measured the CC biometry in T2-weighted imaging and DTI for the remainder of the patient cohort.

### 2.4. Stastistical Analysis

We conducted our analysis without stratifying cases by fetal sex, based on the results of a previous study indicating that there are no statistically significant differences in CC biometry between the sexes [36].

Inter-observer analyses were conducted among three independent observers, in addition to intra-observer analyses for one observer. The interclass correlation coefficient (ICC) was used to assess the agreement between the three observers and the single observer with themselves [35]. The expected ICC was above 0.85 and to achieve a 95% confidence interval (CI) of 0.2, a minimum of 24 participants were required with three observers.

ICC and Bland–Altman plots were used in order to assess the agreement between the biometry measurements found in the T2-weighted sequence and DTI, respectively [37]. ICC values were interpreted based on Koo and Li as ICC ≤ 0.50 poor agreement, 0.50 < ICC < 0.75 moderate agreement, 0.75 < ICC < 0.90 good agreement, and ICC ≥ 0.90 excellent agreement [35]. Statistical analysis was performed using SPSS, IBM Corp. (version 28).

## 3. Results

**Figure 2:**
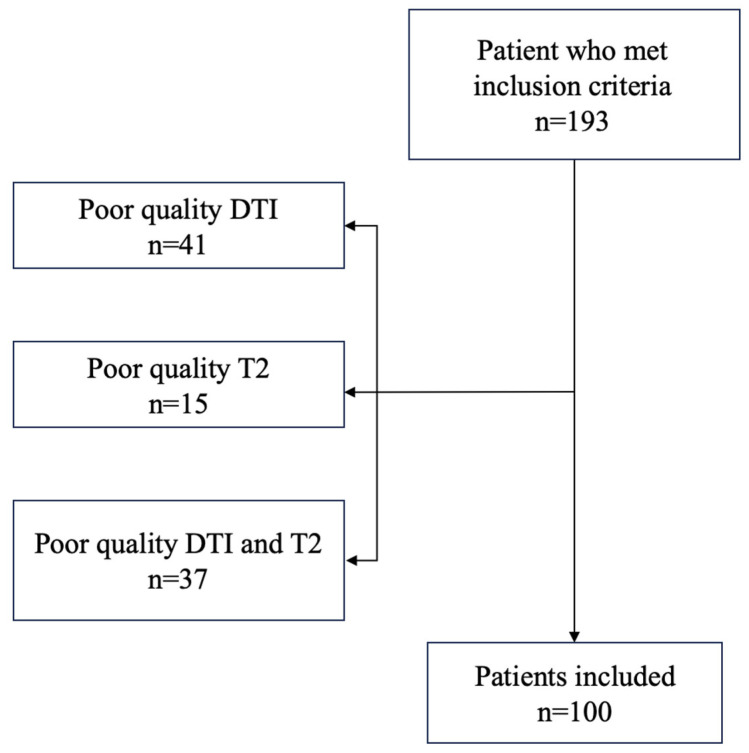
Flow chart of the study.

We identified 193 patients who met our initial inclusion criteria. An additional exclusion was conducted based on the quality of the image (Figure 2).

The quality of the image was defined by the ability to discern the end of the genu and the end of the splenium, thereby facilitating the most accurate measurement of the CC biometry possible. Of those, forty-one patients had poor quality DTI images but adequate T2-weighted image quality, fifteen patients had poor quality T2-weighted images but adequate DTI image quality, and thirty-seven patients had both poor-quality T2-weighted images and DTI images.

Our final sample size for measuring the biometry of the CC was 100. The mean and median gestational age when conducting the imaging was 32.93 ± 1.83 and 32.5 weeks, respectively (range 29–38 weeks). The indications for fetal MRI among the patients are described in Table 1, and all cases were determined to be healthy. The most common indication for the MRI examination was maternal CMV infection during pregnancy in 32 of 100 fetuses. An additional 11 patients were sent for MRI due to a small head circumference, 7 had ventricular asymmetry, 6 had undetermined genetic findings, and 6 had heart defects. Furthermore, five mothers were infected with the Parvo virus during pregnancy, three of which exhibited fetal anemia. Another four suffered from intrauterine growth restriction, two had bilateral club foot, two exhibited polyhydramnios, two had a short CC on US, and two were exposed to toxoplasma. Lastly, 21 fetuses had other indications for MRI including risks for choanal atresia, maternal Roaccutane treatment at the start of the pregnancy, fetal polydactyly, CC malformation visualized on US, hypoplastic vermis, Blake’s pouch cyst, pontocerebellar hypoplasia, oligohydramnios, maternal bacteremia, CC checkup, and concerns for other anatomical malformations.

### 3.1. Inter-Observer and Intra-Observer Agreement

Each observer measured thirty samples independently. There was excellent reliability (absolute agreement) among the three observers in measuring the biometry of the CC in DTI (ICC = 0.904, 95% CI = 0.815–0.952). There was moderate agreement among three observers in measuring the biometry of the CC in T2 (ICC = 0.719, 95% CI = 0.556–0.842). When ICC was calculated between two observers, excellent reliability was measured in both DTI and T2 (ICC = 0.963, 95% CI = 0.925–0.982; ICC = 0.876, 95% CI = 0.763–0.937, respectively), (Table 2).

The intra-observational analysis also exhibited excellent reliability when measuring biometry in DTI and T2 (ICC = 0.967, 95% CI = 0.933–0.984; ICC = 0.942, 95% CI = 0.884–0.971, respectively), (Table 2).

### 3.2. Biometry Measurements in T2-Weighted Imaging Compared with DTI

**Figure 3:**
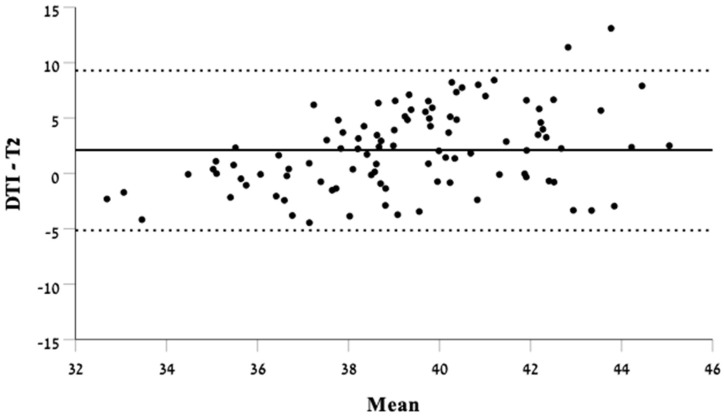
Bland–Altman plot demonstrating the difference in biometry measurements in DTI and T2-weighted sequence.

When comparing biometry measurements in DTI with T2-weighted imaging, we found a poor agreement between the two techniques (ICC = 0.290, 95% CI = 0.071–0.476). The Bland–Altman analysis depicts the lack of agreement along the mean lengths measured of the CC (Figure 3).

## 4. Discussion

In this retrospective cross-sectional study, we found poor agreement between CC biometry measurements in DTI and T2-weighted images. The findings were statistically significant. Based on the Bland–Altman analysis, it appears that the lack of agreement is inconsistent, with no clear tendency for one technique to consistently produce longer or shorter measurements than the other.

To the best of our knowledge, this is the first study comparing the performance of DTI in the evaluation of the fetal CC in normal subjects with the performance of standard T2-weighted imaging. The results of this study are important because the true gold standard for evaluating fetal anatomy typically relies on autopsy findings and postnatal imaging, which are not relevant in many clinical contexts [38,39]. Consequently, despite its limitations, MRI has emerged as the best available tool for assessing fetal corpus callosum (CC) integrity.

Insight into the roles of novel technologies in clinical practice may improve clinical assessments and thus, patient outcomes. More importantly, in the world of fetal health, the precision of available diagnostic tools carries exceptional implications in decision-making of how and whether to proceed with the pregnancy and to prepare parents for the needs of their future child [40]. Thus, it is crucial to understand whether the visualization of fetal anatomical features, such as the CC, is comparable between the tools used in the current protocol and newer technologies that are yet to be fully incorporated into standard care. Previous studies have assessed the contribution of DTI in cases of fetal CC abnormalities. Millischer et al. assessed 33 isolated cases of fetuses with short corpus callosum (SCC) using DTI. The results of the study demonstrated that DTI helped differentiate between types of SCC as a sign of CC dysplasia or simply as a deviation from typical fetal CNS development [28]. In a similar study, Corroenne et al. assessed DTI parameters in SCC compared with cases of normal CC and showed that DTI provided additional differentiation between groups based on alterations of the white matter microstructure [34].

Several technical issues may explain the lack of agreement between the two techniques. Firstly, it is challenging to ensure that the fetal orientation in the T2-weighted sequence slice precisely matches the fetal orientation in the DTI image. Even minor differences in the orientation of the fetal brain can lead to minor and major discrepancies in the length measured. Additionally, it is important to note that in our study, 41 subjects exhibited good quality T2-weighted sequence images, but poor quality DTI images compared to 15 cases of poor quality T2-weighted sequence images with acceptable DTI quality. This may suggest that DTI is more sensitive to fetal movement in utero, posing a potential limitation to this technique. Several studies evaluating the incorporation of DTI in fetal imaging have highlighted the fact that DTI is particularly susceptible to participant movement, leading to artifacts in the images and misalignment with one another [30,41]. In the case of fetal MRI, DTI must overcome both maternal motion, due to respiration, and involuntary fetal bulk movement. The sensitivity of DTI lies not only in the water diffusion motion that achieves the intended contrast but also in general bulk movements that may even disrupt entire slices [42]. It is possible that using motion compensation sequences to cope with the motion in three dimensions in the evaluation of DTI could mitigate the difference in agreement between DTI and T2 weighted sequence. The disadvantage of this technique is the extended scan time to allow for numerous loops; however, this is manageable in the context of fetal scanning as there is no need for breath-holding or sedation [41].

Our study has several limitations. Firstly, the morphology of the CC was not included in the comparison, which may have given more insight into the agreement between the two techniques. Additionally, although the observers exhibited excellent reliability results for measuring the CC biometry, they were not experienced radiologists. In future research, it may be more optimal for the observers to have a previous background in fetal MRI assessment. Lastly, the images in DTI were not corrected for motion disturbances which may have impacted the results. Future studies should assess ways to overcome the susceptibility to motion; for example, measuring biometry using reconstructed 3D images as opposed to two-dimensions and novel tools utilizing artificial intelligence to correct for motion disturbances [41,43].

Our research introduces fresh insights into the comparison of DTI and T2-weighted imaging for assessing fetal CC in healthy fetuses. Given the absence of a definitive gold standard or clear guidelines for evaluating fetal CC biometry, our study raises questions about the preferred technology and highlights the necessary additional considerations when assessing fetal participants.

## 5. Conclusions

In conclusion, our study demonstrated poor agreement between DTI and MRI T2-weighted imaging for fetal CC biometry, indicating that DTI’s advantages over conventional imaging techniques remain unclear in this context and raising questions about its clinical relevance in fetal CC evaluation. Although the combination of T2-MRI and DTI is currently being investigated in some clinical settings, more comprehensive studies are required to establish its applicability and efficiency in routine practice in this field. The implications of this research are far-reaching because comparing these modalities is essential to determining whether a combined approach offers a previously unrecognized diagnostic advantage, ultimately leading to better-informed clinical practices.

## Figures and Tables

**Table 1:** Descriptive data of the indications for fetal brain MRI.

Indication	Count	Percentage (%)
CMV Seroconversion	32	32%
Small Head Circumference	11	11%
Ventricular Asymmetry	7	7%
Genetic Findings	6	6%
Heart Defect	6	6%
Parvo Virus Infection with or without Fetal Anemia	5	5%
IUGR	4	4%
Bilateral Club Foot	2	2%
Polyhydramnios	2	2%
Short CC	2	2%
Toxoplasma Exposure	2	2%
Other	21	21%

Outlines the indications of fetal MRI for each fetus. CMV, cytomegalovirus; CC, Corpus Callosum; IUGR, Intrauterine Growth Restriction.

**Table 2:** Inter-observer and intra-observer agreement for fetal CC biometry measurements with DTI and T2-weighted sequences.

	ICC	95% CI	Significance
**Inter-observer agreement** **(2 observers)**			
CC biometry with DTI (mm)	0.963	0.925–0.982	<0.001
CC biometry with T2 (mm)	0.876	0.763–0.937	<0.001
**Intra-observer agreement**			
CC biometry with DTI (mm)	0.967	0.933–0.984	<0.001
CC biometry with T2 (mm)	0.942	0.884–0.971	<0.001

Representation of the reliability assessment through inter-observer agreement with two observers and intra-observer agreement with a single observer for fetal CC biometry measurements in DTI and T2-weighted sequence. ICC, inter class correlation co-efficient; CC, corpus callosum; DTI, diffusion tension Imaging; T2, T2-weighted sequence.

## Data Availability

The data presented in this study are available on request from the corresponding author. The data are not publicly available due to privacy matters.
